# Urticaria by neurogenic switching of gastroesophageal chemical-infective inflammation: a phenomenon that should always be evaluated in suspected multiple drug hypersensitivity

**DOI:** 10.1186/2045-7022-4-S3-P26

**Published:** 2014-07-18

**Authors:** Fabio De Bartolomeis, Alfonso Savoia, Ernesto Aitella, Carlo Sacerdoti, Adele Parlato, Carmen Palmieri, Corrado Astarita

**Affiliations:** 1 Aggregate Postgraduate School of Allergy & Clinical Immunology of Second University of Naples & Univ, Department of Internal and Experimental Medicine, Italy; 2 Second University of Naples, Department of Internal and Experimental Medicine, Italy

## Background

We report a case of acute urticaria by suspected multiple drug hypersensitivity that has been probably caused by cutaneous neurogenic switching of a chemical-infective neurogenic inflammation induced by a gastroesophageal reflux disease (GERD) with an associated infection by Helicobacter Pylori (HP).

## Methods/results

Recently we evaluated a 48 years old woman for a long lasting suspected multiple drug hypersensitivity. Patient's medical story was characterized by a proved reflux esophagitis, HP infection, and GERD symptoms that were persistent for over 8 years. During the previous 5 years, different treatments of HP infection based on Amoxicillin, Levofloxacin, and Clarithromycin had induced transient mild urticaria within 30 minutes after the first oral intake of drugs. Despite 20 mg/day of Pantoprazole, the patient was still suffering from GERD symptoms. She was non atopic as total serum IgE were low and skin tests for a lot of common inhaled and ingested allergens resulted negative. Skin tests with Ampicillin, Amoxicillin, Cefaclor, other types of Cephalosporins, Clarithromycin, and Levofloxacin were negative at 20 minutes and 72 hours. Moreover, serum specific IgE vs. Ampicillin, Amoxicillin, Cefaclor, and Penicillins were negative. A series of oral challenge was performed. Challenges with increasing oral doses of Amoxicillin (cumulative dose of 385 mg) and Levofloxacin (cumulative dose of 185 mg) induced transient urticaria. When same challenges were repeated, respectively after 2 and 3 weeks of treatment with Pantoprazole 40 mg/day, which induced remission of GERD symptoms, they resulted negative. The patient successfully completed eradication of HP infection with Pantoprazole 80 mg/day for 2 weeks, associated with the sequential intake of Amoxicillin and then of Levofloxacin plus Tinidazole. We have observed, and successfully treated, 4 similar cases in the last 8 months and a lot of GERD-associated, apparently "idiopathic", urticarias in the last 3 years.

## Conclusions

Transient urticarial reactions associated with intake of oral drugs often remain unexplained. GERD and HP infection should be investigated when evidences of immune-mediated mechanisms are lacking. As reported in literature, the neurogenic switching can explain as allergic/non-allergic inflammatory stimuli (infectious agents, chemicals, other) at one site (gastroesophageal tract, in our case) can lead to a neurogenic inflammation at a distant site (skin, in our case).

